# Variance among Public Health Agencies’ Boil Water Guidance

**DOI:** 10.3201/eid3108.250208

**Published:** 2025-08

**Authors:** Megan Dorris, Shanna Miko, Jasen M. Kunz, Vincent R. Hill

**Affiliations:** Epidemic Intelligence Service, Atlanta, Georgia, USA (M. Dorris); Centers for Disease Control and Prevention, Atlanta (M. Dorris, S. Miko, J. Kunz, V. Hill)

**Keywords:** Drinking water, water microbiology, disinfection, temperature, viruses, bacteria

## Abstract

We reviewed boil water guidance from 5 public health agencies and noted differences in boil definition, duration, and elevation adjustment. Publishing evidence-based models could clarify the scientific rationale, promote consensus, and minimize likelihood of incomplete water treatment or excess use of limited fuel resources during emergencies and in backcountry settings.

On September 27, 2024, Hurricane Helene brought strong winds and historic rainfall to the southeastern United States. Powerful flood waters, falling trees, and landslides left millions with limited access to electricity and fuel (*1*). Damage to hundreds of miles of distribution pipes risked contaminating the drinking water supply with disease-causing microorganisms. In response, drinking water utilities issued boil water advisories, affecting >1.8 million persons for days to weeks (*2*).

Boiling is an identifiable target that does not require a thermometer and occurs at 100°C (212°F) at sea level. Although boiling cannot remove suspended particulate matter, disease-causing microbes begin to die or deactivate as water temperature rises, losing their ability to cause illness (*3*). Consequently, boiling is a simple and effective way to disinfect drinking water in emergency situations, during water main breaks or low pressure events in drinking water distribution systems, or in backcountry settings (*4*–*10*). At higher elevations, where water boils at a lower temperature, some guidance recommends longer boil times (*5*,*7*,*8*). However, boiling water requires fuel, a resource often limited in emergency situations and backcountry settings. Variance in boil water guidance from public health agencies might leave the public weighing thorough water treatment against conserving limited fuel supplies.

## The Study

We compared boil water guidance from the US Centers for Disease Control and Prevention drinking water advisories webpage (*5*) and Yellow Book (*6*), US Environmental Protection Agency (EPA) (*7*), Health Canada (*8*), US Department of Homeland Security Ready.gov (*9*), and World Health Organization (WHO) (*10*,*11*) (Table). Our goal was to identify variances in boil water guidance, explore potential reasons for differences, and describe opportunities for future research to support the development of universally consistent guidelines.

**Table:** Comparison of guidance from 5 public health agencies for study of variance among public health agencies’ boil water guidance*

Agency	Target endpoint	End point definition	Duration at endpoint	Elevation adjustment	Cooling guidance
CDC drinking water advisories webpage (*5*)	Full rolling boil	Not defined	1 min	3 min at elevations above 1,981 m (6,500 ft)	Allow boiled water to cool before you use it
Yellow Book (*6*)	Boiling	Not defined	1 min, if fuel supplies are adequate	No adjustment needed at common terrestrial travel elevations.	Not addressed
EPA (*7*)	Rolling boil	Not defined	1 min	3 min at elevations 1,524 m (5,000 feet)	Let water cool naturally
Health Canada (*8*)	Rolling boil	A vigorous boil, where bubbles appear at the center and do not disappear when the water is stirred	1 min	2 min at elevations above 2,000 m (6,562 ft)	The water should then be cooled
Ready.gov (*9*)	Rolling boil	Not defined	1 min	Not addressed	Let the water cool before drinking
WHO (*11*)	Rolling boil	Not defined	No additional time after reaching rolling boil	No adjustment at high elevation	Cool naturally, without the addition of ice

*CDC, US Centers for Disease Control and Prevention; EPA, US Environmental Protection Agency; WHO, World Health Organization.

Each guidance document includes boiling as an endpoint, with most guidance further specifying rolling boil (Table). Health Canada provides a definition to identify rolling boil, and Yellow Book recommends a full minute of boiling to “account for user variability in identifying boiling points.” We speculated that issuing conservative recommendations out of caution might contribute to differences in guidance. Clearly and consistently defining the target endpoint could decrease user variability and enable agencies to recommend less cautious boil times. A concerted effort among public health organizations to set microbial-based targets and a universal health metric, reinforcing the scientific rationale behind water-boiling safety measures, would lay critical groundwork for a unified boil water guidance framework, as would conducting meta-analyses of time–temperature microbial inactivation. Study results from such investigations could inform recommendations for heat time, potential elevation affects, and how the cooling period factors into guidance.

During pasteurization, thermal inactivation of bacteria, viruses, and protozoa begins slowly at temperatures well below boiling point and accelerates as temperatures rise (*3*). Safety specialists perform pasteurization studies at 60°C–85°C (140°F–185°F) in a variety of foods and beverages and measure pathogen inactivation in log reductions. Such studies typically report a ≥3 log (≥99.9%) reduction of most enteric pathogens in times ranging from 1 second to 30 minutes (*3*,*11*). However, few studies document log reductions achieved at or near boiling point or the corresponding timing. Data from pasteurization studies therefore inform estimates of how long water should boil before it is considered potable.

After boiling, cooling time also can contribute to thermal inactivation of pathogens. The EPA, Ready.gov, and WHO recommend letting water cool naturally (Table). During this cooling time, the water would be above pasteurization temperatures longer than other cooling methods, such as refrigerating or adding ice, which could cause recontamination. WHO advises that water may be removed from the heat source immediately after reaching a rolling boil, whereas other agencies recommend maintaining a rolling boil for 1 minute. No guidance specifies whether cooling is included in heat-time estimates of pathogen inactivation.

During pasteurization, minimum pathogen reduction requirements called log-reduction targets (LRTs) are based on initial quantity of pathogen present and the risk the pathogen poses to human health. As for pasteurization standards, boil water guidance aims to make water safe for drinking and cooking (i.e., potable), not sterile (*6*–*8*,*10*). Setting pathogen-specific LRTs for drinking water is difficult because the level of source water contamination is often unknown. In emergency situations and backcountry settings, available water sources may be visibly turbid, requiring higher LRTs to make the water potable. Higher temperatures, longer thermal time, or water filters may be required to achieve greater LRTs, highlighting the importance of achieving a true rolling boil. The use of different LRTs may contribute to variation across boil water guidance. Estimating minimum, pathogen-specific LRTs for potable water could increase transparency of boil water guidance and foster discussion among agencies.

Pathogens require different minimum exposure levels to cause disease and have distinct illness and death rates (*10*). From a microbial perspective, health officials generally consider water potable when pathogens are reduced below the disease-causing threshold. For instance, whereas bacterial spores are highly heat resistant and may survive boiling, they rarely cause human disease and are considered a tolerable risk in drinking water (*6*).

Health outcome metrics help estimate tolerable risk levels. The EPA estimates a tolerable risk as a level of exposure causing <1 illness/10,000 persons/year (*12*). WHO uses disability-adjusted life-years and defines tolerable burden of disease as 10^−6^ disability-adjusted life-years/person/year (*10*). Adopting a universal health metric for water safety standards could assist in establishing consistent LRTs for waterborne pathogens.

Researchers have used time-temperature models based on pasteurization data to illustrate pathogen inactivation time to specific LRTs. Although data >85°C (185°F) is often lacking, those models could be adapted to estimate pathogen-specific LRTs at boiling or near-boiling temperatures (Figure) and highlight pathogens lacking data points. Quantifying reductions for certain pathogens, such as norovirus and rotavirus, is challenging because of the lack of reliable methods for measuring viable units, but pathogens with equal or greater thermal resistance can be used as proxies to estimate inactivation (*10*).

**Figure:**
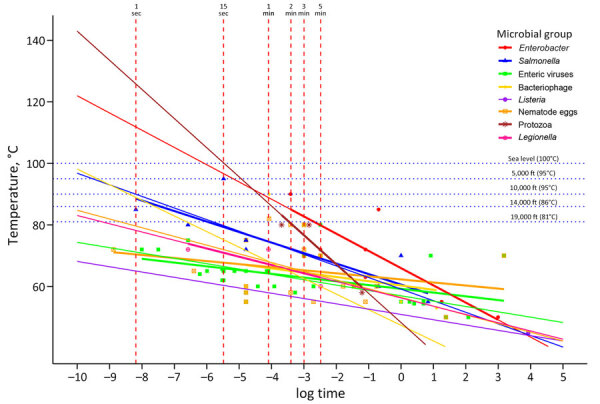
Linear model of boiling time required to reduce pathogen levels, by elevation, for study of variance among public health agencies’ boil water guidance. Model estimates time to achieve 3–5 log reductions of specified microbial groups (*3*,*11*). At boiling point temperatures, all listed pathogens achieve the reductions in ≤1 minute, from sea level to 10,000 feet. Incorporating more data points at boiling or near-boiling temperatures could enhance accuracy. Models using pathogen-specific log reduction targets may provide more precise time estimates.

Existing time–temperature models could also help evaluate how elevation affects boil water recommendations. For every 150-m (492-ft) increase in elevation, the boiling point of water decreases by ≈0.5°C (0.9°F). In Asheville, North Carolina, USA, at 640 m (2,100 ft) elevation, water boils at 98°C (207.6°F), whereas in Denver, Colorado, USA, at 1,585 m (5,200 ft) elevation, water boils at ≈94°C (201°F). This inverse relationship between elevation and boiling point contributes to differences in boil water guidance at high elevations. Heat inactivation models could estimate when pathogen-specific LRTs are achieved at these lower boiling points, potentially incorporating cooling time to account for additional inactivation—and possibly providing support for discounting boil time adjustment for higher elevations.

Yellow Book and WHO guidance state that all disease-causing pathogens, except bacterial spores, are inactivated at boiling temperatures. Developing and publishing models to illustrate this guidance could clarify the scientific basis for decisions across agencies, identify gaps in pathogen inactivation data, and encourage informed discussion.

## Conclusions

Extreme weather events can damage water systems, utilities, and roadways, limiting access to safe drinking water and fuel. To address this concern, public health agencies have published boil water guidance to educate the public on preparing safe drinking water during emergencies. Variances in boiling time and elevation adjustment across these guidelines may cause confusion, however, potentially hindering efforts to ensure water safety or leading to extra fuel use.

Although the concept of boiling water to ensure potability is straightforward, developing evidence-based guidance is complex. Adopting a consistent definition of a rolling boil, publishing analyses or models based on pathogen-specific LRTs supported by health outcome metrics, and incorporating cooling time into models could enhance clarity. This approach could illustrate the scientific rationale behind current guidance, encourage informed multi-agency discussion, and create opportunities to build consensus on boil water recommendations.

## References

[1] State of North Carolina Office of State and Budget Management. Hurricane Helene damage needs assessment: Hurricane Helene recovery recommendations. December 13, 2024. [cited 2024 Dec 11]. https://www.osbm.nc.gov/hurricane-helene-dna/open

[2] Phillis MAJ, Peterson B. A week after Helene hit, thousands still without water struggle to find enough [cited 2024 Dec 11]. https://apnews.com/article/hurricane-helene-asheville-north-carolina-water-737bd51b1351315dc28522e36c425f18

[3] Espinosa MF, Sancho AN, Mendoza LM, Mota CR, Verbyla ME. Systematic review and meta-analysis of time-temperature pathogen inactivation. Int J Hyg Environ Health. 2020;230:113595. 10.1016/j.ijheh.2020.113595 32814236

[4] Centers for Disease Control and Prevention, US Environmental Protection Agency, American Water Works Association, Association of State and Territorial Health Officials, Association of State Drinking Water Administrators, National Environmental Health Association. Drinking water advisory communication toolbox. Updated 2016 [cited 2024 Dec 9] https://www.cdc.gov/water-emergency/media/pdfs/2024/08/dwact-2016.pdf

[5] Centers for Disease Control and Prevention. Drinking water advisories: an overview. [cited 2024 Dec 9]. https://www.cdc.gov/water-emergency/about/drinking-water-advisories-an-overview.html

[6] Backer H, Hill V. Water disinfection. 2023. In: CDC Yellow Book 2024: health information for international travel [cited 2024 Dec 9]. https://wwwnc.cdc.gov/travel/yellowbook/2024/preparing/water-disinfection

[7] US Environmental Protection Agency. Emergency disinfection of drinking water [cited 2024 Dec 9]. https://www.epa.gov/ground-water-and-drinking-water/emergency-disinfection-drinking-water

[8] Health Canada. Guidance for issuing and rescinding boil water advisories in Canadian drinking water supplies. 2015 [cited 2024 Dec 9]. https://www.canada.ca/en/health-canada/services/publications/healthy-living/guidance-issuing-rescinding-boil-water-advisories-canadian-drinking-water-supplies.html

[9] US Department of Homeland Security. Water [cited 2024 Dec 9]. https://www.ready.gov/water

[10] World Health Organization. Guidelines for drinking-water quality: 4th edition, incorporating the 1st addendum. 2017 [cited 2024 Dec 9]. https://www.who.int/publications/i/item/9789241549950 28759192

[11] World Health Organization. Boil water. 2015 [cited 2024 Dec 11]. https://iris.who.int/handle/10665/155821

[12] Sharvelle S, Ashbolt N, Clerico E, Holquist R, Levernz H, Olivieri A. Risk-based framework for the development of public health guidance for decentralized non-potable water systems. Proc Water Environ Fed. 2017;8:3799–809. 10.2175/193864717822158189

